# Cost effectiveness of different screening strategies for gestational diabetes mellitus screening: study protocol of a randomized community non-inferiority trial

**DOI:** 10.1186/s13098-019-0493-z

**Published:** 2019-12-18

**Authors:** Fahimeh Ramezani Tehrani, Gulf Study Cooperative Research Group, Samira Behboudi-Gandevani, Mehrandokht Abedini, Masoud Soleymani-Dodaran, Davood Khalili, Farshad Farzadfar, Farhad Hoseinpanah, Farzad Hadaegh, Majid Valizadeh, Farahnaz Torkestani, Zahra Abdollahi, Marzieh Bakhshandeh, Razieh Bidhendi Yarandi, Mehdi Zokaee, Farzam Bidarpour, Mehdi Javanbakht, Iraj Nabipour, Mohammad Ali Mansournia, Ensieh Nasli Esfahani, Afshin Ostovar, Fereidoun Azizi, Abbas Najari, Abdolmohhamad Khajeian, Azita Anaraki, Fariba Ghazaghi, Forouzan Lahouni, Forouzandeh Kalantari, Hossein Fallah, Khadije Kordi, Lotfollah Saed, Mahsa Norooozzadeh, Maryam Farahmand, Marzieh Rostami Dovom, Mehdi Hedayati, Mehdi Mehdizade, Mina Amiri, Mohammad Hassan Lotfi, Mohammad-Esmaeil Motlagh, Mozhgan Bashghareh, Nosrat Zamanipour, Parvin Mirmiran, Saeid Sadeghian Sharif, Saeid Shahraz, Samareh Khari, Sedigheh Alishahi, Shole Shahgheibi, Sima Nazarpour, Yadollah Mehrabi, Zahra Ghaedmohammadi

**Affiliations:** Reproductive Endocrinology Research Center, Research Institute for Endocrine Sciences, Shahid Beheshti University of Medical Sciences, No 24, Parvane Street, Yaman Street, Velenjak, P.O.Box: 19395-4763, Tehran, Iran

**Keywords:** Cost-effectiveness, Gestational diabetes, Screening, Perinatal outcome

## Abstract

**Background:**

There is lack of ideal and comprehensive economic evaluations of various GDM strategies. The aim of this study is to the compare efficacy and cost-effectiveness of five different methods of screening for gestational diabetes mellitus (GDM).

**Methods:**

This study is a randomized community non-inferiority trial among 30,000 pregnant women in five different geographic regions of Iran, who were randomly assigned to one of the five GDM screening methods. All first trimester pregnant women, seeking prenatal care in governmental health care systems, who met our eligibility criteria were enrolled. The criteria suggested by the International-Association-of-Diabetes-in-Pregnancy-Study-Group, the most intensive approach, were used as reference. We used the non-inferiority approach to compare less intensive strategies to the reference one. Along with routine prenatal standard care, all participants were scheduled to have two phases of GDM screening in first and second-trimester of pregnancy, based on five different pre-specified protocols. The screening protocol included fasting plasma glucose in the first trimester and either a one step or a two-step screening method in the second trimester of pregnancy. Pregnant women were classified in three groups based on the results: diagnosed with preexisting pre-gestational overt diabetes; gestational diabetes and non-GDM women. Each group received packages for standard-care and all participants were followed till delivery; pregnancy outcomes, quality of life and cost of health care were recorded in detail using specific standardized questionnaires. Primary outcomes were defined as % birth-weight > 90th percentile and primary cesarean section. In addition, we assessed the direct health care direct and indirect costs.

**Results:**

This study will enable us to compare the cost effectiveness of different GDM screening protocols and intervention intensity (low versus high).

**Conclusion:**

Results which if needed, will also enable policy makers to optimize the national GMD strategy as a resource for enhancing GDM guidelines.

*Trial registration* Name of the registry: Iranian Registry of Clinical Trials. Trial registration number: IRCT138707081281N1. Date of registration: 2017-02-15. URL of trial registry record: https://www.irct.ir/trial/518

## Background

Gestational diabetes (GDM) defined as hyperglycaemia at any time during pregnancy at levels below those that occurring in overt diabetes [1]. It is one of the most common glycemic disorders during pregnancy with occurrence of 1–28% of all pregnancies [2–5], along with the increased rate of obesity and advanced maternal age is rising in prevalence [6]. It is well documented that GDM is associated with both short as well as long term higher rates of adverse feto-maternal and neonatal outcomes [7–11]. From an obstetrical perspective, evidence shows that treatment of GDM is effective in reducing the risk of many of the important adverse pregnancy outcomes [12–14].

Despite the globally accepted importance of screening for and treating GDM [13], screening strategies, testing methods and even diagnostic optimum glycemic thresholds for GDM remained much controversy for decades and no international consensus has been yet established [15]. In addition, the former screenings were mainly performed to prevent adverse maternal outcomes compared to neonatal complications. Considering this, use of different tests and criteria will impact the prevalence of women diagnosed with GDM [5], and could also impact poor pregnancy outcomes [16, 17]. There is also much controversy about milder forms of GDM. For which, the associations of mild GDM with adverse pregnancy outcomes are not completely understood; there is ongoing debate about the benefits of treating mild GDM and the impact on health care costs [18–21].

The Hyperglycemia and Adverse Pregnancy Outcomes (HAPO) study demonstrated that hyperglycemia at levels below those previous recommended thresholds for GDM were associated with adverse maternal and neonatal outcomes; hence, the International Association of Diabetes in Pregnancy Study Group (IADPSG) introduced new cutoffs for the 2-hour (2 h) oral glucose tolerance test (OGTT) in GDM screening and diagnosis [22]. Besides, at present, a 3-h 100 g diagnostic test is used predominantly in the United States and some other areas, whereas much of the world uses the 75 g, 2-h OGTT [5]. At present, there is little information regarding the sensitivity and specificity of these test, and hence the relative clinical effectiveness of the two-steps of the 1-h 50-g glucose challenge test (GCT) following 3-h 100 g oral glucose tolerance (OGTT) diagnostic test and the one-step OGTT approaches in the same population. However using the IADPSG criteria, two to threefold more women qualified for a diagnosis of GDM, potentially adding to the costs of care of the already large number of pregnant women [23–25].

With both increased prevalence and adopting lowering of the thresholds for diagnosis, the healthcare cost of GDM can be expected to rise proportionately. It follows that the debate as to whether or not a benefit exists in the treatment of GDM assumes even greater importance now than in the past. However, since in most countries, resources are inevitably scarce, healthcare interventions should be evaluated for their impact the on cost as well as effectivity on clinical outcomes [26]. Moreover, while not recognizing that GDM is associated with adverse pregnancy outcomes, over-diagnosis may lead to psychological stress, unnecessary treatments and impaired quality of life [27–29].

There is lack of ideal and comprehensive economic evaluations of various GDM strategies; the majority of existing cost-effective analyses are based on decision analysis modelling not real data, limited obtained from randomized clinical trials that documented controversial results [20, 30–38]. In addition most studies have been conducted in well-developed high-income countries which obviously have more developed healthcare systems than low and middle-income countries, where gestational diabetes has the highest prevalence. According to a WHO report, global and local decision-making regarding GDM strategies are challenging due to the lack of optimum economic evaluations of various GDM screening protocols, making it difficult to validated implement any national recommendations from a health economic perspective [31]. Since resources are unavoidably scarce, national health care interventions should be assessed for their impact on costs as well as on clinical outcomes; the most highly recommended practice is that economic evaluation should be an integral part of randomized clinical trials [39]; each population needs to adopt its community specific guidelines [40].

In this ongoing randomized community-field non-inferiority trial, we aimed to compare the cost-effectiveness of five different pre-defined GDM screening protocols, both one and two step, using different fasting plasma glucose thresholds to ascertain the optimum GDM screening protocol.

## Materials and methods

### Research questions and objectives

This study is being performed to provide real data collected from an unbiased population trial for assessment of the following hypothesis: (i) the prevalence of GDM when using the less intensive GDM screening strategies is not more than obtained using the IADPSG criteria. (ii) The pre-specified primary outcomes in less intensive GDM screening strategies are not worse than those obtained using IADPSG criteria. (iv) The cost of health care using less intensive GDM screening strategies is not higher than incurred using IADPSG criteria. (v) The numbers needed to treat (NNT) to prevent one primary outcome in less intensive strategies are the same as those obtained using IADPSG criteria.

The cost of prevention for one primary outcome in less intensive strategies is the same as that for IADPSG criteria.

According to our research hypothesis, primary outcomes hence are: percentage of birth weight > 90th percentile and primary cesarean section. Secondary outcomes are prevalence of neonatal hypoglycemia, birth weight < 10th percentile, neonatal admission to the intensive care unit, shoulder dystocia and birth trauma including fracture of clavicle and brachial plexus injury, intrauterine fetal death, preeclampsia and preterm labor, neonatal hyperbilirubinemia and hypocalcemia. In addition, the study will assess the direct health care costs including prenatal clinic visits, obstetrician visits, endocrinologist visits, dietician visits, blood glucose monitoring equipment, laboratory test cost, pharmacotherapy, additional fetal well-being assessments and hospitalization as well as indirect cost of productivity loss and charges to the family including traveling, food substitution, mother time off paid work, and partner time off work.

### Overall study design

This is a randomized community-field trial including five GDM screening strategies in a parallel group design. Recruitment of the participants took place between September 2016 and January 2019 in 1015 health centers in 25 selected cities of five provinces of Iran.

All pregnant women < 14 weeks of gestation, who received prenatal care from governmental health care systems were eligible for enrollment, except where the following specific exclusion criteria prevented this: Maternal age < 18 years, preexisting diabetes, date of last menstrual period not certain, no ultrasound estimation from 6 to 14 weeks of gestational age available, chronic hypertension, asthma or currently receiving treatment with oral glucocorticoids, β-blockers, oral β-mimetics, Dilantin, or antiretroviral agents and past history of bariatric surgery.

All participants received standard prenatal care recommended by the American College of Obstetricians and Gynecologists (ACOG) [41]. Moreover, participants were scheduled to have two phases of GDM screening in the first and second trimesters of pregnancy, based on the pre-specified protocol for GDM screening, selected for each city.

At each prenatal visit, standardized questionnaires were administered to document prenatal as well as other data needed for research by trained midwives.

### Sample size calculation

Based on previous studies, we assumed that the primary event rate of macrosomia to be equal to 10% for all groups with no difference. To obtain a statistical power of 85% with a 1-sided type one error of 0.005 (considering multiple comparisons) approximately 4700 patients per group are needed to show the non-inferiority of more intensive compared to lower intensive strategies with a marginal difference of 0.03. With a design effect of 0.001 (for cluster sampling) and loss to follow-up of 11%, sample size reached to 5200 in each group [42].

In addition, superiority analyses will be designed to show that one screening strategy is superior to another after non-inferiority has been demonstrated.

### Randomization and allocation

Initially all provinces of Iran were categorized to five stratum based on their geographic location (North, East, West, South, and Center of Iran) and one province in each stratum were randomly selected; then, the list of the cities located in each province were provided. Since the socioeconomic status in the center of provinces may differ from other cities, in the second phase, all cities in each province were classified in two clusters of center of the province and other cities. At the end, four cities were randomly selected from the list of other cities in each province.

**Fig. 1:**
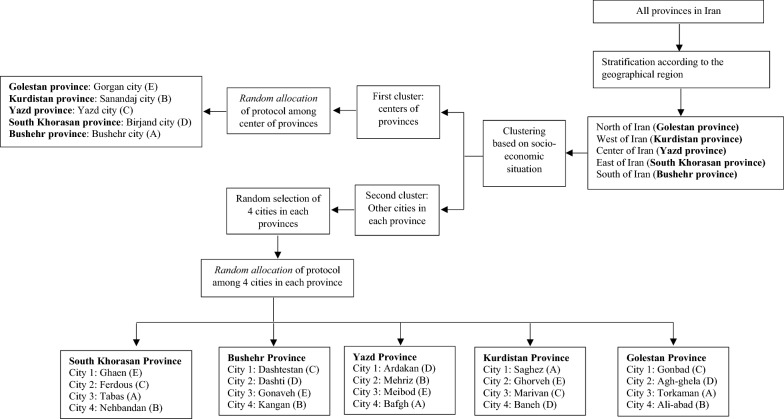
Randomization and allocation of study

For allocation of protocols, in the cluster of the provincial centers, five different protocols were randomly allocated to each provincial center. Also, in the cluster of other cities, four other cities in each province were randomly allocated to the rest of the protocols (Fig. 1). Sample size for each city was estimated through probability proportional to size (PPS), defined by number of live births of the cities.

### Intervention

Following the approval of this study, the study procedure was released as a guideline to all the selected cities. In this respect, workshops were conducted in each city to introduce the study protocol and train the caregivers and study staff accordingly. Dieticians, obstetricians, internal medics, laboratory technicians and endocrinologists in each province were invited to a scientific workshop to harmonize and coordinate the follow ups and treatment of GDM patients. Scientific teams with specialists and executive members conducted visits every 2 months. A telegram channel was developed for daily online communication of scientific members and executive members at both provincial and city levels to answer questions and solve any problems encountered.

Along with routine standard prenatal care, all pregnant women was screened for GDM based on the pre-specified protocol assigned to each city. In this respect, early screening of GDM was conducted in the first trimester of pregnancy, using fasting plasma glucose (FPG) from venous sample with the specific threshold based on each screening protocol; based on the results of those screening tests, pregnant women were classified in to three groups: (i) diagnosed with preexisting pre-gestational overt diabetes; (ii) gestational diabetes and (iii) non-GDM women. In addition, at 24–28 weeks of gestation, those not previously known to have diabetes (overt or gestational), were screened again for GDM based on pre-specified protocol criteria assigned to that city. All study participants were followed till delivery and pregnancy and neonatal outcomes and health cost were recorded in detail. Definitions of various protocols for screening are presented in Table 1.

**Table 1:** Definitions of various protocols for screening of gestational diabetes mellitus

Protocol	First trimester	Second trimester		
	Diagnostic criteria for GDM	Method for GDM screening	Diagnostic threshold of test	Diagnostic criteria
A	92 mg/dL < FPG > 126 mg/dL	One step with 2-h 75 g OGTT	Fasting ≥ 92 mg/dL 1 h ≥ 180 mg/dL 2 h ≥ 153 mg/dL	GDM is defined as any of the given plasma glucose values are met or exceeded
B	100 mg/dL < FPG > 126 mg/dL	One step with 2-h 75 g OGTT	Fasting ≥ 92 mg/dL 1 h ≥ 180 mg/dL 2 h ≥ 153 mg/dL	GDM is defined as two or more of the given plasma glucose values are met or exceeded
C	100 mg/dL < FPG > 126 mg/dL	One step with 2-h 75 g OGTT	Fasting ≥ 92 mg/dL 1 h ≥ 180 mg/dL 2 h ≥ 153 mg/dL	GDM is defined as any of the given plasma glucose values are met or exceeded
D	92 mg/dL < FPG > 126 mg/dL	Two steps with 50 g GCT—1 h following 3-h 100 g OGTT	50 g GCT:	GDM is defined as if two or more of the given plasma glucose values in 100 g OGTT are met or exceeded
			BS-1 h: ≥ 140 mg	
			100 g OGTT:	
			Fasting ≥ 95 mg/dL	
			1 h ≥ 180 mg/dL	
			2 h ≥ 155 mg/dL	
			3 h ≥ 140 mg/dL	
E	100 mg/dL < FPG > 126 mg/dL	Two steps with 50 g GCT—1 h following 3-h 100 g OGTT	50 g GCT:	GDM is defined as if two or more of the given plasma glucose values in 100 g OGTT are met or exceeded
			BS-1 h: ≥ 140 mg	
			100 g OGTT:	
			Fasting ≥ 95 mg/dL	
			1 h ≥ 180 mg/dL	
			2 h ≥ 155 mg/dL	
			3 h ≥ 140 mg/dL	

In the first trimester overt diabetes is defined as FPG ≥ 126 mg/dL

*FPG* fasting plasma glucose, *GCT* glucose challenge test, *OGTT* oral glucose tolerance test

Each group received packages of standard care based on their health status. In this respect, non-GDM pregnant women received routine standard care recommended by the American College of Obstetricians and Gynecologists (ACOG) 2013 [41]. Moreover, pregnant diabetic patients received specific prenatal and diabetic care, recommended by the American College of Obstetricians and Gynecologists (ACOG) 2013 [43] and the American Diabetes Association (ADA) 2016 [44].

### Summary of management of Gestational Diabetes in Pregnancy

After diagnosis of GDM, treatment was initiated with medical nutrition therapy, physical activity, and weight management and blood glucose monitoring to achieve the targets recommended by ADA guideline 2016 [44] including fasting, 95 mg/dL, 1-h postprandial, 140 mg/dL or 2-h postprandial, 120 mg/dL. Medical nutrition therapy for GDM will be individually planed for participants by the dietitian. The food plan provides enough calorie intake to promote fetal/neonatal and maternal health, achieve glycemic goals, and promote appropriate gestational weight gain, based on the Dietary Reference Intakes (DRI) recommendation including a minimum of 175 g carbohydrate, a minimum of 71 g protein, and 28 g fiber [44].

**Fig. 2:**
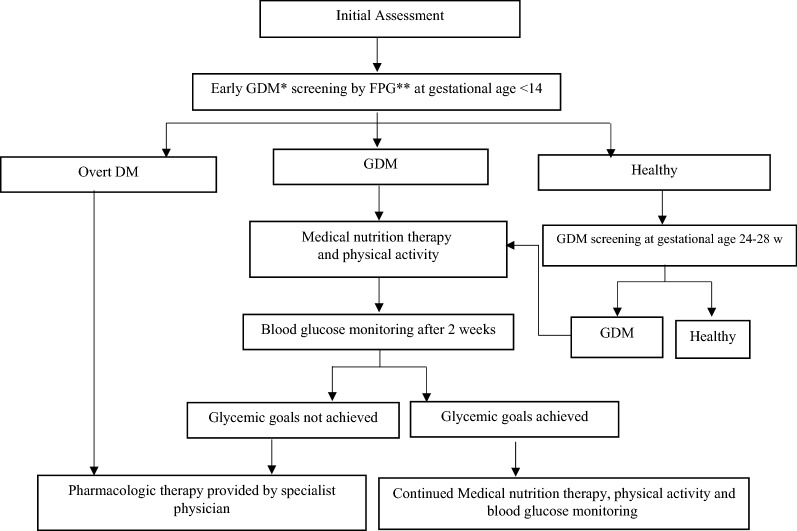
Flow chart of screening and management of Gestational Diabetes in Pregnancy. *GDM: gestational diabetes mellitus; **FPG: fasting plasma glucose

If women did not achieve glycemic goals within 2 weeks, pharmacologic therapy will be offered by specialized physicians including obstetricians, internists or endocrinologists at the second level of the healthcare delivery system. Insulin is the first-line agent recommended for treatment of GDM. Self-monitoring of blood glucose (SMBG) was used for achieving and maintaining therapeutic goals in insulin-treated patients. The frequent use of capillary blood glucose tests of SMBG was scheduled four times a day, fasting, 2-h after breakfast, lunch and dinner or if the patients had hypoglycemic symptoms for at least 2 weeks. After achieving the therapeutic target, SMBG was performed two times a day. In addition, if women decline insulin therapy, metformin will be offered as an alternative or adjunct to insulin after clarifying the harms and benefits of metformin therapy for patients [44] (Fig. 2).

### Data collection

Data were collected from participants at scheduled time points (Table 2) using pre-specified questionnaires and clinical and para clinical exams by trained midwives. Moreover, data on neonatal mortalities that occurred after hospital discharge were collected at 4 weeks post-partum by telephone and subsequent reviews of medical records.

**Table 2:** Outlines of periodic assessments of study participants

	Method or sample used	> 14 week	14–19 week	20–23 week	24–30 week	31–34 week	35–37 week	38 week	39 week	40 week	Birth	28 days after birth
Maternal^a,b^												
Past medical, reproductive and obstetrics history												
Weight	Calibrated scale	^a^	^a^	^a^	^a^	^a^	^a^	^a^	^a^	^a^		
Height	Stadiometer	^a^										
Blood pressure (systolic, diastolic)	Calibrated mercury sphygmomanometer	^a^	^a^	^a^	^a^	^a^	^a^	^a^	^a^	^a^		
Fundal height	Measuring tape		^a^	^a^	^a^	^a^	^a^	^a^	^a^	^a^		
Fetal heart rate			^a^	^a^	^a^	^a^	^a^	^a^	^a^	^a^		
Fetal ultrasound		^a^			^a^		^a^					
FPG	Venous sample	^a^										
OGTT-75 g or GCT following OGTT-100 g	Venous sample				^a^							
Quality of life	Questionnaire		^a^	^a^	^a^	^a^		^a^				
Drug adherence	Questionnaire		^a^	^a^	^a^	^a^		^a^				
GDM treatment satisfaction	Questionnaire		^a^	^a^	^a^	^a^		^a^				
Cost-effectiveness	Questionnaire		^a^	^a^	^a^	^a^		^a^			^a^	
Feto-maternal outcomes^c^											^a^	
Neonatal^a,b^												
C-peptide	Cord sample										^a^	
Weight	Calibrated baby scale										^a^	
Recumbent length	Infantometer										^a^	
Head circumference	Measuring tape										^a^	
Blood glucose^e^	Heel-stick sample										^a^	
Neonatal outcomes^d^											^a^	^a^

^a^Data collected from routine and expert scans that occur during the time points

^b^If GDM or other complication were diagnosed, subsequent additional visits, measurements and standard treatment were performed

^c^Feto-maternal outcomes continuously recoded include abortion, gestational hypertension, pre-eclampsia/eclampsia, preterm birth, instrumental delivery, primary cesarean section, polyhydramnios, oligohydramnios, premature rupture of membrane, placenta Previa, placenta abruption, postpartum hemorrhage, wound and incision infection

^d^Neonatal outcomes include shoulder dystocia, intrauterine growth restriction, macrosomia, Apgar score, neonatal hypoglycemia, neonatal hypocalcemia, neonatal hyperbilirubinemia, polycythemia, neonatal intensive care unit admission, neonatal care unit admission, Respiratory distress syndrome, congenital anomaly, neonatal asphyxia, intrauterine fetal death, perinatal death, Erb–Duchenne palsy, birth trauma, neonatal sepsis

^e^Measured for high risk groups

### Questionnaires

1. *Prenatal questionnaire* This comprehensive questionnaire includes two sections: 1—contains the past medical, reproductive, obstetrics, and gynecological history, completed only at first prenatal visit 2—focuses on current pregnancy information and this part was completed at each prenatal visit during pregnancy (Additional file 1: PART 1: Prenatal Care Form).
2. *Delivery, postpartum and neonatal questionnaire* This questionnaire contains the details of delivery and its methods and any adverse maternal–fetal/neonatal outcomes (Additional file 1: PART 2. Childbirth and New-born Report Form).
3. *Quality of life questionnaire* The Iranian version of 36-item short form health survey questionnaire (SF-36) [45–48] was used to measure the physical and mental components of health-related quality of life. The SF-36 included 36 items with 8 subscales; physical functioning, role limitations due to physical problems, bodily pain, general health perceptions, vitality, social functioning, role limitations due to emotional problems and perceived mental health. This questionnaire was completed monthly for all GDM patients since the time of diagnosis. Also, it were done for 5% of non-GDM pregnant women visited from the first visit for prenatal care (Additional file 1: PART 3. 36-Item Short Form Survey Instrument).
4. *Cost-effectiveness questionnaire* This questionnaire included 50 items with three subscales: (i) self-purchased health care, (ii) travel costs for making return visit(s) to health care and (iii) time costs of travelling and attending health care center. Effectiveness was measured in terms of quality adjusted life years (QALYs), using the EQ-5D 3L questionnaire completed by participants at the follow up time points. It includes five questions, each assessing one of five dimensions of the health related quality of life (Mobility, Self-Care, Usual Activities, Pain/Discomfort and Anxiety/Depression). Each of these dimensions has to be answered on a 3-level scale (no problems, some or moderate problems, and extreme problems). The scales are scored from 1 (no problem) to 3 (extreme problem) in each question; and finally the score digits are placed together to yield a 5-digit code for the health status of each patient (Additional file 1: PART 4. Cost effectiveness Form).

### Maternal anthropometric, clinical, and laboratory assessments

Weight was measured to the nearest 100 g using digital scales while the participants were minimally clothed, without shoes. Height was measured to the nearest 0.5 cm, in a standing position without shoes, using a tape measure, while shoulders were in normal alignment. Body mass index (BMI) was calculated as weight (kg) divided by height squared (m^2^). After a 15-min rest in the sitting position, two measurements of systolic and diastolic blood pressure (SBP and DBP) were taken on the right arm, using a standardized mercury sphygmomanometer (calibrated by the Iranian Institute of Standards and Industrial Researches); the mean of the two measurements was considered as the participant’s blood pressure.

Plasma glucose were measured on the day of blood collection. A blood sample was drawn between 7:00 and 9:00 AM from all study participants, after 8 to 10 h overnight fasting. For the 75-g OGTT-82.5 g of glucose monohydrate solution (equivalent to 75 g anhydrous glucose), for the 50 g glucose challenge test (GCT)-55 g of glucose monohydrate solution (equivalent to 50 g anhydrous glucose) and for the 100-g OGTT-100 g of glucose monohydrate solution (equivalent to 110 g anhydrous glucose) were administered orally to subjects and plasma glucose was measured, using an enzymatic colorimetric method with glucose oxidase; inter- and intra-assay coefficients of variation were less than 2.3%. Analyses were performed using Pars Azmon kits (Pars Azmon Inc., Tehran, Iran) using the Selectra 2 auto-analyzer (Vital Scientific, Spankeren, Netherlands).

### Neonatal anthropometric, clinical, and laboratory assessments

Neonatal anthropometric and clinical measurement were measured by trained staff. Birth weight was measured without diapers using a calibrated digital baby scale (SECA model 334; SECA Corp., Hamburg, Germany) to the nearest 1 gr, within an hour after delivery. Recumbent length was measured to nearest 0.1 cm from the top of the head to the sole of the feet using an infantometer (Easy-Glide Bearing Infantometer, Perspective Enterprises). Head circumference (HC) was measured at the largest occipito-frontal diameter and the measurement was rounded to the nearest 0.25 cm. The largest of three consecutive measurements was recorded.

In this respect, two measurements were obtained, and if results differed by > 10 g for weight and 0.5 cm for length or head circumference, a third measurement were taken. The average of the two or three measurements was used for final analysis.

According to the national Iranian guidelines, all newborns were exclusively breastfed early after delivery. Infants were either screened for hypoglycemia 1–2 h after birth before a feeding based on the presence of defined risk factors including maternal GDM/overt DM, birth weight > 90th percentile, maternal BMI > 30, birth weight < 10th percentile, early preterm birth less than 34 weeks of gestation, perinatal acidosis, 5-min Apgar score of 0–3, failure of breastfeed and sepsis.

In this respect, blood glucose levels were measured using heel-stick sampling at 1, 3, 6, 12, and 24 h after birth before a feeding. Additional blood glucose measurements were performed in case of hypoglycemia or clinical symptoms including sweating, weak or high-pitched cry, feeding difficulties, poor sucking, tremors, hypothermia, irritability, lethargy/stupor, hypotonia, seizures, apnea, grunting or tachypnea or cyanosis. Using point-of-care testing, glucose was measured with the glucose oxidase method (Pars Azmon Inc., Tehran, Iran).

Cord serum C-peptide sample, as the index of fetal β-cell function, was collected at the time of delivery in a subsample of 1000 participants with different screening protocol. Samples collected were centrifuged for 10 min at 3000 rpm, stored at − 80 °C and transferred to central laboratory. C-peptide were determined with ELISA method (Mercodia AB, Uppsala, Sweden); the inter- and intra-assay coefficient of variation were < 2.3% and 1.5%, respectively.

The need for other assessments, such as serum bilirubin or imaging tests were determined based on clinical indications.

### Definition of study outcomes

Outcomes of study were defined as follows: Macrosomia/large for gestational age (LGA) was defined as birth-weight > 4000 g and/or fetal-weight > 90th percentile for a given gestational age [49] using ultrasound biometry for estimating the fetal-weight and multinational World Health Organization (WHO) fetal growth chart for defining the percentile. Primary cesarean section was defined as the cesarean deliveries out of all births to women who had not had a previous cesarean delivery [50]; abortion refers to a termination of a pregnancy either natural or induced before the completion of 20 weeks of gestation. Polyhydramnios is defined as excess accumulation of amniotic fluid with 4-quadrant amniotic fluid index (AFI) more than 24 cm or a single maximum vertical pocket more than 8 cm [51]. Oligohydramnios refers to decreased amniotic fluid volume relative to gestational age with AFI less than 24 cm or a single maximum vertical pocket less than 8 cm [52]. Intrauterine growth restriction (IUGR)/fetal growth restriction was defined as fetal-weight less than the 10th percentile for gestational age [53] using ultrasound biometry for estimating the fetal-weight and multinational World Health Organization (WHO) fetal growth chart for defining the percentile. Small size for gestational age (SGA) refers to birth-weight less than the 10th percentile for gestational age [53, 54] using gender specific WHO weight-for-age chart for defining the percentile. Hypoglycemia was defined as plasma glucose concentration < 47 mg/dL in the first 48 h after delivery [55, 56]; hyperbilirubinemia was determined by value greater than the 95th percentile for any given point after birth [57]; Gestational hypertension was defined as a systolic pressure of ≥ 140 mmHg or a diastolic pressure of ≥ 90 mmHg taken on two occasions, at least 4 h apart [58, 59]; Preeclampsia was defined as an elevation in blood pressure ≥ 140 mmHg systolic or ≥ 90 mmHg diastolic on two occasions at least 4 h apart after 20 weeks of gestation in a women with a previously normal blood pressure and proteinuria ≥ 300 mg per 24 h urine collection or protein/creatinine ratio greater than or equal to 0.3 or dipstick reading of 1+ and more if other quantitative methods were not available. In the absence of proteinuria, new-onset hypertension with the new onset of any of the thrombocytopenia, renal insufficiency, impaired liver function, pulmonary edema and cerebral or visual symptoms [59]; preterm birth was defined as when birth occurs between 20 and 37 weeks of pregnancy [60]; shoulder dystocia was defined clinically, where providers are required to provide additional obstetric maneuvers when gentle downward traction has failed to affect the delivery of the shoulders [61] and birth trauma was defined as brachial plexus palsy or clavicular, humeral, or skull fracture. Mild GDM is defined as: a fasting glucose level of > 92 and < 100 mg per decilitre in 1st trimester of pregnancy and only one glucose measurement exceeding from established thresholds for 2-h 75gOGTT as follows: FPG > 92 mg/dL, 1-h plasma glucose >  180 mg/dL, 2-h plasma glucose >  53 mg/dL at the 24–28 weeks of gestation.

### Data cleaning and missing data

The following minimal data must be available for women to be included in the analysis of pregnancy outcomes: Completed enrollment forms and questionnaire, completed results of GDM screening, type of delivery, birth weights and clear status of exclusionary criteria.

Missing values will be managed using appropriate imputation methods. Outliers will be identified using graphical tools including boxplot and/or Model-based methods like Chauvenet’s criterion and Dixon’s Q test [62, 63].

### Data analysis

To illustrate distribution of the data, appropriate descriptive statistics such as measures of central tendency, index of dispersion and percentiles will be reported along with normality assumption testing through Kolmogorov-Smirnoff test. Maternal, neonatal and obstetric outcomes of the 4 less intensive screening strategies with IADPSG criteria will be compared using parametric or non-parametric statistical tests, where applicable.

In addition, based on the type of outcome variables, Generalized Linear Models (GLMs) with different link function such as linear, count or binary will be applied. Stepwise method with P-value < 0.2 will be used to identify significant confounding variables and estimate adjusted measures of interests. Moreover, longitudinal modeling through Generalized Estimating Equation (GEE) analysis approach will be conducted and to calculate Number Needed to Treat (NNT), the Linear GLM model will be applied as well. Since this is a cluster randomized trial, cluster effect in analysis will be considered.

### Cost-effectiveness analysis (CEA)

A cost-effectiveness analysis, comparing 4 less intensive screening strategies with IADPSG criteria will be conducted on an intention-to-treat basis by estimating various parameters including Quality-adjusted life years (QALYs), incremental cost-effectiveness ratio (ICER), and incremental net benefit (INB). To estimate mean cost in each treatment group, regression models will be used. General linear models (GLM) with appropriate variance functions e.g. gamma, Poisson, etc. and link will be used to identify the relationship between treatment allocation and costs after adjusting for minimization and the appropriate prognostic covariates at baseline (e.g. Baseline EQ-5D score). To estimate the incremental effect of the treatment indicator variable, recycled predictions will be used [64].

A sensitivity analysis will be conducted to assess how sensitive the cost-effectiveness results are to variation in key parameters including cost.

### Bayesian and Markov Modeling

**Fig. 3:**
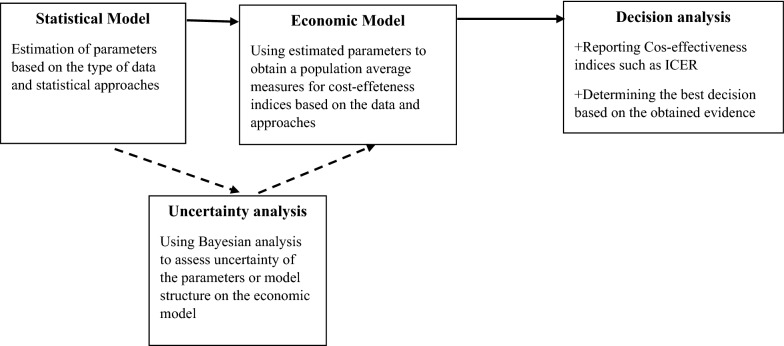
A schematic illustration of the process of health economic evaluation

Bayesian Cost Effectiveness Modeling (BCEM) will be used to overcome the complexity of the relationships linking a suitable measure of clinical benefit (e.g. quality-adjusted life years) and the associated costs. Simplifying assumptions, such as normality of the underlying distributions, are usually not granted, particularly for the cost variable, which is significantly skewed distributions. In addition, individual-level data sets are often characterized by the presence of structural zeros in the cost variable [65–67]. Bayesian models will be used to account for the presence of excess zeros in a distribution and have been applied in the context of cost data (Fig. 3).

Markov model will be used to extrapolate the results of the trial beyond the follow up, which will eventually provide longer-term cost-effectiveness. Markov decision processes (MDPs) are a powerful and appropriate technique for modelling medical decision. MDPs are most useful in classes of problems involving complex, stochastic and dynamic decisions like medical treatment decisions, for which they can find optimal solutions [68]. Physicians will always need to make subjective judgments about treatment strategies, but mathematical decision models can provide insight into the nature of optimal choices and guide treatment decisions [69]. Markov models can be used to describe various health states in a population of interest, and to detect the effects of various policies or therapeutic choices. In addition, we will apply decision tree analysis and then apply probabilistic approach.

All data analysis will be conducted using R (Version 2.2.2) and TreeAge (Version 13) softwares.

### Approval and ethical considerations

This trial has been approved and funded by the National Institute for Medical Research Development under Grant Agreement No IR.NIMAD.REC.1394.013. Funding source had no involvement in the study. The protocol was approved by the national ethics committee of the National Institute for Medical Research Development (Approval number: IR.NIMAD.REC.1394.013). In addition, the Iranian Ministry of Health and Medical Education (MoHME) approved the study protocol and pre specified GDM modalities were made available to all those provinces as mandatory guidelines. This field trial has been registered in Iranian Registry of Clinical Trials (Trial Registration: IRCT138707081281N1).

## Discussion

At present, there is a lack of international consensus about the diagnosis of gestational diabetes. Screening strategies, testing methods and even diagnostic optimum glycemic thresholds for GDM remain the subject of considerable debate. Although gestational diabetes mellitus is a recognized marker for an increased risk of subsequent diabetes, its clinical significance with respect to its various definitions and various adverse pregnancy outcomes has not been clearly elucidated. Women with severe gestational diabetes and highly elevated fasting plasma glucose levels apparently are at an increased risk for adverse pregnancy outcomes if treatment is not provided, yet the association of milder forms of gestational diabetes with such outcomes remains unclear. Despite the HAPO study having provided valuable evidence of the association of maternal blood glucose with adverse pregnancy outcomes, it is worth noting that HAPO study was a purely observational study that conducted in western countries.

Considering the fact that majority of births annually occur in low- and low–middle income countries with high prevalence of GDM and limited resources [5], the cost-effectivity of this definition needs to be re-evaluated in other communities; the present study will hopefully provide such information from an eastern Mediterranean region. Moreover there is little information comparing the clinical efficacy, utility and feasibility of the two step GDM screening test and a 3-h oral glucose tolerance test (GTT) and the one step oral glucose tolerance test (OGTT) approaches, our study will provide comprehensive data on this comparison in the same population.

According to a WHO report, global and local decision making regarding GDM strategies are challenging due to the lack of optimum economic evaluations of various GDM screening protocols; as a result our study will provide the data needed for each community to adopt its specific GDM screening guidelines according to the reasonable cost for prevention of the adverse short and long term effects of GDM.

The limitations of our study of course should be addressed. Since specific questionnaires for evaluation of QOL and drug adherence in patients with GDM were not available, general questionnaires was used. In addition, we did not use the central reference laboratory for all of our measurement except C-peptide. Since homogeneity of laboratory procedures are essential to the success the study, we used standardized procedures in all provinces including local training of field center laboratory personnel, using a common protocol for measurement of glucose; using of standard equipment and supplies; monthly external quality controls for each laboratory. Moreover, glycosylated hemoglobin A1c (HbA1c) measurements were not available in our study.

## Conclusions

Results which if needed, will also enable policy makers to optimize the national GMD strategy as a resource for enhancing GDM guidelines.

## Supplementary information

**Additional file 1:** PART 1: Prenatal Care Form; PART 2. Childbirth and New-born Report Form; PART 3. 36-Item Short Form Survey Instrument; PART 4. Cost effectiveness Form.

## Data Availability

The datasets used and analyzed during the current study are available from the corresponding author on reasonable request.

## Abbreviations

GDM: gestational diabetes mellitus
IADPSG: the International Association of Diabetes in Pregnancy Study Group
OGTT: oral glucose tolerance test
WHO: World Health Organization
ACOG: American College of Obstetricians and Gynecologists
FPG: fasting plasma glucose
ADA: American Diabetes Association
DRI: Dietary Reference Intakes
QALYs: quality adjusted life years
AFI: amniotic fluid index
SGA: small size for gestational age
IUGR: intrauterine growth restriction
GLMs: Generalized Linear Models
GEE: Generalized Estimating Equation
INB: incremental net benefit
ICER: incremental cost-effectiveness ratio
BCEM: Bayesian Cost Effectiveness Modeling
