# Supplementary material for: Cost effectiveness of different screening strategies for gestational diabetes mellitus screening: study protocol of a randomized community non-inferiority trial
Source: Diabetol Metab Syndr. 2019 Dec 18;11:106. doi: 10.1186/s13098-019-0493-z (PMC6921504; doi:10.1186/s13098-019-0493-z)
Supplement: Supplementary file 1 — Additional file 1: PART 1: Prenatal Care Form; PART 2. Childbirth and New-born Report Form; PART 3. 36-Item Short Form Survey Instrument; PART 4. Cost effectiveness Form. [file 13098_2019_493_MOESM1_ESM.docx]

**Additional file**

**PART 1. Prenatal Care Form**

**Identification information**

| Name of University / faculty: | Name of city: | Name of health center: | Identification number: |
| --- | --- | --- | --- |
| First name of pregnant woman: | Last name of pregnant woman: | Birth date: | National code: |
| First Name of the questioner: | Last name of questioner: | Questioner code: | Date: |
| Address: | | Phone or mobile number: | Job: |

**Section 1: history**

| **Current pregnancy status** | | | |
| --- | --- | --- | --- |
| Number of pregnancies:  Number of abortion: | Number of deliveries:  Number of vaginal deliveries:  Number of Cesarean: Section deliveries: | Current pregnancy  Singleton □ twins □ | Number of children:  Number of live children:  Number of dead children: |
| Date of last delivery: | Date of last menstrual period (LMP): | Gestational age (based on the ultrasonography):  Date: | Gestational age (by week) at completion of medical record:  Unknown gestational age: |
| Estimated date of confinement (EDC): | Height: | Weight: | Body mass index (BMI): |
| Parent kinship : Yes □ No □ | | | |

| **Previous pregnancies and births** | | | |
| --- | --- | --- | --- |
| □ Preeclampsia/eclampsia | □ Gestational diabetes | □ Multiple pregnancy | □ Uterine atony |
| □ Operative vaginal delivery (forceps or vacuum) | □ Rapid delivery | □ Placental abruption | □ Mole |
| □ Ectopic pregnancy | □ Recurrent miscarriage | □ Late term abortion | □ Uterus surgery |
| □ Severe post-partum hemorrhage (PPH) | □ Post term birth | □ Preterm birth | □ Intrauterine growth restriction (IUGR) |
| □ Abnormal neonate or abortion due to abnormality | | □ Neonatal death |  |
| □ Neonatal weight < 2500 | □ Neonatal weight > 4000 | □ Still birth | |

| **Diseases / anomalies in current pregnancy** | | | |
| --- | --- | --- | --- |
| □ Heart disease  Type: | □Thyroid disease  Type: | □ Kidney disease  Type: | □ Connective tissue disease Type: |
| □ Gastrointestinal disease  Type: | □ Minor thalassemia woman □ husband □ | □ Chronic hypertension | □ Epilepsy |
| □ Asthma | □ Coagulation disorder | □ Tuberculosis | □ Genital abnormalities |
| □ Diabetes | □ History of breast cancer | □ Hepatitis | □ Psychiatric disorder |
| □ Multiple sclerosis | □ HIV+/AIDS | □ Iron deficiency anemia | □ Sickle cell anemia |
| □ Thrombophilia / Thromboembolism | □ Pregnancy hypertension | □ Gestational diabetes  Treated only with diet □ treated with diet + drug □ | |
| □ Preeclampsia | □ History of menstrual disorders | □ Hirsutism | □ History of polycystic ovaries morphology (PCOM) |
| □ Dyslipidemia | □ Medical allergy | □ Type of drugs used: | □ Smoking |
| □ Other (please mention details) | | | |

| **Family history** | |
| --- | --- |
| Genetic disorder in a wife or her husband or abnormalities in one of the first-degree relatives of the couple (father, mother, brother, child) | Yes □ No □ |
| Type 2 diabetes in first-degree relatives (father, mother, sister, brother, child) | Yes □ No □ |
| Chronic hypertension in first-degree relatives (father, mother, sister, brother, child) | Yes □ No □ |

**Section 2: Results of laboratory tests and sonography**

| **First trimester pregnancy tests** | **Request date** | **Sampling date** | **Requested test** | **Result** | **Interpretation of laboratory test result** |
| --- | --- | --- | --- | --- | --- |
|  |  |  | FBS |  | Healthy □ Gestational diabetes □ Overt diabetes □ |

| **Third trimester pregnancy tests**  **(24 to 28 weeks)** | **Request date** | **Sampling date** | **Type of test** | **Requested test** | | **Result** | **Interpretation of laboratory test results** |
| --- | --- | --- | --- | --- | --- | --- | --- |
|  |  |  | One step | OGTT-75g glucose | FBS |  | Healthy □  Gestational diabetes |
|  |  |  |  |  | 1h |  |  |
|  |  |  |  |  | 2h |  |  |
|  |  |  | Two step | GCT-50g glucose | |  | Healthy □ diabetic □ Next step □ |
|  |  |  |  | OGTT-100g glucose | FBS |  | Healthy □  Gestational diabetes □ |
|  |  |  |  |  | 1h |  |  |
|  |  |  |  |  | 2h |  |  |
|  |  |  |  |  | 3h |  |  |

| **Screening of first trimester of pregnancy** | | | | **Results** |
| --- | --- | --- | --- | --- |
| **Request date** | **Screening date** | **Gestational age at screening** | **Nuchal Translucency (NT)** | Low risk □  High risk □ |
|  |  |  |  |  |
| **Screening of third trimester of pregnancy (if needed)** | | | | |
| **Request date** | **Sampling date** | **Gestational age at screening** | **Result of blood test** | **Results** |
|  |  |  |  | Low risk □  High risk □ |
| **Amniocentesis** | | | | |
| **Request date** | **Screening date** | **Gestational age at amniocentesis** | | **Results** |
|  |  |  |  | Normal □  Abnormal □ |

**Results of sonography**

| No. | Date | Gestational age | | Results of sonography | | Biophysical profile results | | | | |
| --- | --- | --- | --- | --- | --- | --- | --- | --- | --- | --- |
|  |  |  |  |  |  | Item | Score | | | |
|  |  |  |  |  |  |  | 0 | 1 | 2 | total |
|  |  | According to the LMP | According to the sonography | Placenta (position and grade) |  | NST |  |  |  |  |
|  |  |  |  | Amniotic fluid (volume) |  | Respiratory movements |  |  |  |  |
|  |  |  |  | Fetal anomaly |  | Fetal movement |  |  |  |  |
|  |  |  |  | Key points |  | Muscle tone |  |  |  |  |
|  |  |  |  |  |  | Amniotic fluid |  |  |  |  |
| No. | Date | Gestational age | | Results of sonography | | Biophysical profile results | | | | |
|  |  |  |  |  |  | Item | Score | | | |
|  |  |  |  |  |  |  | 0 | 1 | 2 | total |
|  |  | According to the LMP | According to the sonography | Placenta (position and grade) |  | NST |  |  |  |  |
|  |  |  |  | Amniotic fluid (volume) |  | Respiratory movements |  |  |  |  |
|  |  |  |  | Fetal anomaly |  | Fetal movement |  |  |  |  |
|  |  |  |  | Key points |  | Muscle tone |  |  |  |  |
|  |  |  |  |  |  | Amniotic fluid |  |  |  |  |
| No. | Date | Gestational age | | Results of sonography | | Biophysical profile results | | | | |
|  |  |  |  |  |  | Item | Score | | | |
|  |  |  |  |  |  |  | 0 | 1 | 2 | total |
|  |  | According to the LMP | According to the sonography | Placenta (position and grade) |  | NST |  |  |  |  |
|  |  |  |  | Amniotic fluid (volume) |  | Respiratory movements |  |  |  |  |
|  |  |  |  | Fetal anomaly |  | Fetal movement |  |  |  |  |
|  |  |  |  | Key points |  | Muscle tone |  |  |  |  |
|  |  |  |  |  |  | Amniotic fluid |  |  |  |  |
| No. | Date | Gestational age | | Results of sonography | | Biophysical profile results | | | | |
|  |  |  |  |  |  | Item | Score | | | |
|  |  |  |  |  |  |  | 0 | 1 | 2 | total |
|  |  | According to LMP | According to the sonography | Placenta (position and grade) |  | NST |  |  |  |  |
|  |  |  |  | Amniotic fluid (volume) |  | Respiratory movements |  |  |  |  |
|  |  |  |  | Fetal anomaly |  | Fetal movement |  |  |  |  |
|  |  |  |  | Key points |  | Muscle tone |  |  |  |  |
|  |  |  |  |  |  | Amniotic fluid |  |  |  |  |

**Note:** If there is an anomaly, mention its type.

**Section 3: Routine evaluation**

|  | | | 6-10 w | 11-15 w | 16-20 w | 21-23 w | 24-30 w | 31-34 w | 35-37 w | 38 w | 39 w | 40 w | 41 w |
| --- | --- | --- | --- | --- | --- | --- | --- | --- | --- | --- | --- | --- | --- |
| Date | | |  |  |  |  |  |  |  |  |  |  |  |
| Gestational age (weeks) | | |  |  |  |  |  |  |  |  |  |  |  |
| Ask | Complications | Spotting/ vaginal bleeding |  |  |  |  |  |  |  |  |  |  |  |
|  |  | Nausea and vomiting |  |  |  |  |  |  |  |  |  |  |  |
|  |  | Rupture of membranes/ fluid leakage |  |  |  |  |  |  |  |  |  |  |  |
|  |  | Pain (head, abdomen, flanks, legs and thighs) |  |  |  |  |  |  |  |  |  |  |  |
|  |  | Rapid pulse rate/ shortness of breath |  |  |  |  |  |  |  |  |  |  |  |
|  |  | Genitourinary disorders |  |  |  |  |  |  |  |  |  |  |  |
|  |  | Reduction / absence of fetal movement |  |  |  |  |  |  |  |  |  |  |  |
|  |  | Skin disorders |  |  |  |  |  |  |  |  |  |  |  |
|  |  | Domestic violence issues |  |  |  |  |  |  |  |  |  |  |  |
|  |  | Trauma |  |  |  |  |  |  |  |  |  |  |  |
|  | Nutrition | Common complaint |  |  |  |  |  |  |  |  |  |  |  |
|  |  | Using pharmaceutical supplements |  |  |  |  |  |  |  |  |  |  |  |
|  |  | Proper nutrition |  |  |  |  |  |  |  |  |  |  |  |
| Measure or examine. | | Weight |  |  |  |  |  |  |  |  |  |  |  |
|  |  | Blood pressure |  |  |  |  |  |  |  |  |  |  |  |
|  |  | Temperature |  |  |  |  |  |  |  |  |  |  |  |
|  |  | Fundal height |  |  |  |  |  |  |  |  |  |  |  |
|  |  | Fetal heart rate |  |  |  |  |  |  |  |  |  |  |  |
|  |  | Edema (Hands, face, legs, coldness of the limbs) |  |  |  |  |  |  |  |  |  |  |  |
| Evaluation result at each visit | | |  |  |  |  |  |  |  |  |  |  |  |

**Section 3: Evaluation of gestational diabetes**

Please provide the following health care if a pregnant woman had gestational diabetes:

A: Time of gestational diabetes diagnosis: Date: Gestational age (weeks):

B: Nutrition education, lifestyle modification and exercise for gestational diabetes: Yes □ No □ Gestational age (w)

C: Result: Blood glucose modification □ High blood sugar and referral □

| **No** | **Date of insulin administration** | **Type and dosage of insulin** | | | | | |
| --- | --- | --- | --- | --- | --- | --- | --- |
|  |  | **Before breakfast** | **After breakfast** | **Before lunch** | **After lunch** | **Before dinner** | **After dinner** |
|  |  |  |  |  |  |  |  |
|  |  |  |  |  |  |  |  |
|  |  |  |  |  |  |  |  |
|  |  |  |  |  |  |  |  |
|  |  |  |  |  |  |  |  |
|  |  |  |  |  |  |  |  |
|  |  |  |  |  |  |  |  |
|  |  |  |  |  |  |  |  |
|  |  |  |  |  |  |  |  |
|  |  |  |  |  |  |  |  |
|  |  |  |  |  |  |  |  |

| **No** | **Date of blood glucose control at gestational diabetes follow ups** | **FBS** | **BS, 2hpp** | **HbA1c** |
| --- | --- | --- | --- | --- |
|  |  |  |  |  |
|  |  |  |  |  |
|  |  |  |  |  |
|  |  |  |  |  |
|  |  |  |  |  |
|  |  |  |  |  |
|  |  |  |  |  |
|  |  |  |  |  |
|  |  |  |  |  |
|  |  |  |  |  |
|  |  |  |  |  |
|  |  |  |  |  |

| **Daily self-monitoring blood glucose (SMBG) form at home*** | | | | | | |
| --- | --- | --- | --- | --- | --- | --- |
| **blood glucose monitoring times per day** | | | | | | |
| **Date** | **Day** | **Fasting** | **2 h after breakfast** | **2h after lunch** | **2h after dinner** | **Blood glucose in abnormal cases** |
|  | Saturday | ✓ |  |  |  |  |
|  | Sunday |  |  | ✓ |  |  |
|  | Monday |  | ✓ |  |  |  |
|  | Tuesday | ✓ |  |  | ✓ |  |
|  | Wednesday |  |  | ✓ |  |  |
|  | Thursday |  |  |  | ✓ |  |
|  | Friday |  | ✓ |  |  |  |
| *This form is to be completed at home by the patients | | | | | | |

**PART 2. Childbirth and Newborn Report Form**

**Identification form**

| Name of University / faculty: | Name of city: | Name of health center: | Identification number: |
| --- | --- | --- | --- |
| First name of pregnant woman: | Last name of pregnant woman: | Birth date: | National code: |
| Name of the questioner: | Last name of questioner: | Questioner code: | Date: |
| Phone or mobile number: | | | Job: |
| Address: | | | |
| Name of Doctor: | | Admission date: time of admission: | |
| Ward: | Room: | Bed: | |

1. **Chief complaint**

| □ initiation onset of labor pains | □ Rupture of membrane | □ Decreased amniotic fluid |
| --- | --- | --- |
| □ Hemorrhage | □ Decreased fetal movement | □ Post term birth |
| □ Planned caesarian section | □ Need to terminate pregnancy due to fetal indications | □ Need to terminate pregnancy due to maternal indications |

1. **Progress of labor**

| Admission date: | | Admission time (h): | |
| --- | --- | --- | --- |
| Duration of delivery phases | First stage: ……… h  Latent phase:…..…h  Active phase:……. h  Transition phase:…h | Time of rupture of membrane | Before 37 weeks of pregnancy……..□  Before beginning of labor pains ………….□  Along with the onset of labor pain.................. □ |
|  | Second stage: ….. min |  |  |
|  | Third stage: ….. min |  | Amniotomy ………□ |
| Use of oxytocin to induction or stimulation of labor pains: Yes □ No □ | | Volume of amniotic fluid: Normal □  Oligohydramnios □ Polyhydramnios □ | |
| Systolic blood pressure □ | | Diastolic blood pressure □ | |

1. **Childbirth**

| Date and time of delivery: | Neonate sex: Female □ Male □ Ambiguous □ |
| --- | --- |
| Delivery Method: Vaginal □ Cesarean □ | |
| Episiotomy: Yes □ No □  If rupture, its degree: 1□ 2 □ 3 □ 4 □  Operative vaginal delivery □ vaginal birth after cesarean □  Shoulder dystocia Yes □ No □ If yes, applied maneuver: …  Has the neonate got any complications? Yes □ No □ | Cause of cesarean section:  Repeated cesarean section □ meconium plug □  Abnormal labor  Fetal distress □  Hypertension, headache, blurred vision, or preeclampsia □ Malpresentations (breech, face, transverse) □ Cephalopelvic disproportion □  Placenta and cord disorders □  Post term labor □ Macrosomia □  Multiple pregnancy □ infertility □  Intra uterine restriction (IUGR) □  History of uterine surgery or uterine anomalies □  Diabetes, impaired GTT □ |

1. **Postpartum care** (The first two hours(

| Estimated postpartum hemorrhage level  The first hour after childbirth: 1 or non □ 2 □ 3 □ 4 □ | |
| --- | --- |
| Uterine atony (second hour after childbirth): 1 or non □ 2 □ 3 □ 4 □ | |
| Perineal status at the time of discharge:  Normal □ hematoma □ disruption of sutures □ | Status of suture at the time of discharge:  Normal □ hematoma □ disruption of sutures □ |

1. **Pregnancy/ birth complication**

| 1. Mild preeclampsia Yes□ No□ 2. Severe preeclampsia Yes□ No□ 3. HELLP syndrome Yes□ No□ 4. Preterm birth Yes□ No□ 5. Premature rupture of membrane Yes□ No□ 6. Placenta previa Yes□ No□ 7. Placenta abruption Yes□ No□ 8. Shoulder dystocia Yes□ No□ 9. Intrauterine growth disturbance Yes□ No□ 10. Macrosomia Yes□ No□ | 1. Failure to progress in labor Yes□ No□ 2. Operative vaginal delivery Yes□ No□ 3. Emergency caesarean section Yes□ No□ 4. Polyhydramnios Yes□ No□ 5. Oligohydramnios Yes□ No□ 6. Post-partum hemorrhage Yes□ No□ 7. Metritis Yes□ No□ 8. Infection of the episiotomy site Yes□ No□ 9. Post-cesarean wound infection Yes□ No□ 10. Abortion Yes□ No□ 11. Other Yes□ No□ |
| --- | --- |

1. **History of neonate at birth**

| Birth date: | Time of delivery (h): |
| --- | --- |
| Neonate sex: Female □ Male □ Ambiguous □ Number of babies □ Birth rank □ Umbilical cord blood sample □ | |
| Anomaly at birth: no anomaly□ Neural tube defect □ Nervous system □ Genital, urinary and kidney system □ Hands and feet □ Down syndrome (trisomy 21) □ Other chromosomal anomalies□ cleft lip / cleft palate □ Cardiovascular □ Musculoskeletal system □ Gastrointestinal tract □ Ears and eyes□ Neck and face □ Other anomalies □  Birth trauma No□ Yes□ Type of anomaly: … | |

1. **Apgar table**

| **Apgar score** | | | | | |
| --- | --- | --- | --- | --- | --- |
| **Sign** | **0** | **1** | **2** | **1st minute** | **2nd minute** |
| **Appearance** | Blue/pale | Extremities blue | All pink |  |  |
| **Pulse** | Absent | < 100 | >100 |  |  |
| **Grimace** | No response | Grimace | Vigorous cough |  |  |
| **Activity (muscle tone)** | Limp | Some flexion/extension | Active motion |  |  |
| **Respiration** | Absent | Slow/irregular | Good cry |  |  |
| **Total score:** | | | |  |  |

1. **Resuscitation table**

| **Minutes** | **1** | **5** | **10** | **15** | **20** |
| --- | --- | --- | --- | --- | --- |
| Oxygen |  |  |  |  |  |
| PPV/NCPAP |  |  |  |  |  |
| ETT |  |  |  |  |  |
| Chest Compressions |  |  |  |  |  |
| Epinephrine |  |  |  |  |  |

1. **Physical examination and evaluations at birth**

| Weight (gr): height (cm): Head circumference (cm): Gestational age (weeks) |
| --- |
| BS: Blood calcium: Total bilirubin: Direct bilirubin: Umbilical C-Peptide: |
| Neonatal jaundice: Yes□ No □ If there is jaundice, please specify treatment: phototherapy□ blood exchange □ |
| Hospitalized in NICU: Yes□ No□ Breastfeeding Yes□ No □ Vomiting Yes □ No□  Fever Yes □ No □ Other: …. |

1. **Neonatal complications**

| 1. Respiratory distress syndrome (RDS) Yes□ No□ 2. Birth trauma Yes □ No □ 3. Fetal anomaly Yes □ No □ 4. Neonatal asphyxia Yes □ No □ 5. Shoulder dystocia Yes □ No□ | 1. Hyperbilirubinemia Yes□ No□ 2. Hospitalized in NICU Yes□ No□ 3. Intra uterine fetal death Yes □ No□ 4. Perinatal mortality Yes □ No□ | 1. Erb's palsy Yes □ No □ 2. Hypoglycemia Yes □ No □ 3. Hypocalcemia Yes□ No□ 4. Polycythemia Yes □ No □ 5. Neonatal infection Yes □ No□ 6. Other: … |
| --- | --- | --- |

**PART 3. 36-Item Short Form Survey Instrument (SF-36)**

**Identification information**

| Name of University / faculty: | Name of city: | Name of health center: | Identification number: |
| --- | --- | --- | --- |
| First name of pregnant woman: | Last name of pregnant woman: | Birth date: | National code: |
| Name of the questioner: | Last name of questioner: | Questioner code: | Date: |
| Phone or mobile number: | | | Job: |
| Address: | | | |

1. In general, would you say your health is:

1 - Excellent

2 - Very good

3 - Good

4 - Fair

5 – Poor

2. Compared to one year ago, how would you rate your health in general now?

1 - Much better now than one year ago

2 - Somewhat better now than one year ago

3 - About the same

4 - Somewhat worse now than one year ago

5 - Much worse now than one year ago

The following items are about activities you might do during a typical day. Does your health now limit you in these activities? If so, how much?

| **Activities** | **Yes,**  **limited a lot** | **Yes, limited a little** | **No, not**  **limited at all** |
| --- | --- | --- | --- |
| 3. Vigorous activities, such as running, lifting heavy objects,  participating in strenuous sports | 1 | 2 | 3 |
| 4. Moderate activities, such as moving a table, pushing a  vacuum cleaner, bowling, or playing golf | 1 | 2 | 3 |
| 5. Lifting or carrying groceries | 1 | 2 | 3 |
| 6. Climbing several flights of stairs | 1 | 2 | 3 |
| 7. Climbing one flight of stairs | 1 | 2 | 3 |
| 8. Bending, kneeling, or stooping | 1 | 2 | 3 |
| 9. Walking more than a mile | 1 | 2 | 3 |
| 10. Walking several blocks | 1 | 2 | 3 |
| 11. Walking one block | 1 | 2 | 3 |
| 12. Bathing or dressing yourself | 1 | 2 | 3 |

During the past 4 weeks, have you had any of the following problems with your work or other regular daily activities as a result of your physical health?

| **Problems as a result of your physical health** | Yes | No |
| --- | --- | --- |
| 13. Cut down the amount of time you spent on work or other activities | 1 | 2 |
| 14. Accomplished less than you would like | 1 | 2 |
| 15. Were limited in the kind of work or other activities | 1 | 2 |
| Had difficulty performing the work or other activities (for example, it took extra effort) | 1 | 2 |

During the past 4 weeks, have you had any of the following problems with your work or other regular daily activities as a result of any emotional problems (such as feeling depressed or anxious)?

| **Problems as any emotional problems** | Yes | No |
| --- | --- | --- |
| 17. Cut down the amount of time you spent on work or other activities |  |  |
| 18. Accomplished less than you would like |  |  |
| 19. Didn't do work or other activities as carefully as usual |  |  |

20. During the past 4 weeks, to what extent has your physical health or emotional problems interfered with your normal social activities with family, friends, neighbors, or groups?

1 - Not at all

2 - Slightly

3 - Moderately

4 - Quite a bit

5 – Extremely

21. How much bodily pain have you had during the past 4 weeks?

1 - None

2 - Very mild

3 - Mild

4 - Moderate

5 - Severe

6 - Very severe
 22. During the past 4 weeks, how much did pain interfere with your normal work (including both work outside the home and housework)?

1 - Not at all

2 - A little bit

3 - Moderately

4 - Quite a bit

5 – Extremely

These questions are about how you feel and how things have been with you during the past 4 weeks. For each question, please give the one answer that comes closest to the way you have been feeling.

How much of the time during the past 4 weeks...

| Question about feel | All of  the  time | Most  of the  time | A good  bit of the  time | Some  of the  time | A little  of the  time | None  of the  time |
| --- | --- | --- | --- | --- | --- | --- |
| 23. Did you feel full of pep? | 1 | 2 | 3 | 4 | 5 | 6 |
| 24. Have you been a very nervous person? | 1 | 2 | 3 | 4 | 5 | 6 |
| 25. Have you felt so down in the dumps that nothing could cheer you up? | 1 | 2 | 3 | 4 | 5 | 6 |
| 26. Have you felt calm and peaceful? | 1 | 2 | 3 | 4 | 5 | 6 |
| 27. Did you have a lot of energy? | 1 | 2 | 3 | 4 | 5 | 6 |
| 28. Have you felt downhearted and blue? | 1 | 2 | 3 | 4 | 5 | 6 |
| 29. Did you feel worn out? | 1 | 2 | 3 | 4 | 5 | 6 |
| 30. Have you been a happy person? | 1 | 2 | 3 | 4 | 5 | 6 |
| 31. Did you feel tired? | 1 | 2 | 3 | 4 | 5 | 6 |

32. During the past 4 weeks, how much of the time has your physical health or emotional problems interfered with your social activities (like visiting with friends, relatives, etc.)?

1 - All of the time

2 - Most of the time

3 - Some of the time

4 - A little of the time

5 - None of the time

How TRUE or FALSE is each of the following statements for you.

| Item | Definitely  true | Mostly  true | Don't  know | Mostly  false | Definitely  false |
| --- | --- | --- | --- | --- | --- |
| 33. I seem to get sick a little easier than other people | 1 | 2 | 3 | 4 | 5 |
| 34. I am as healthy as anybody I know | 1 | 2 | 3 | 4 | 5 |
| 35. I expect my health to get worse | 1 | 2 | 3 | 4 | 5 |
| 36. My health is excellent | 1 | 2 | 3 | 4 | 5 |

**PART 4. Cost effectiveness Form**

| First name of pregnant woman: | Last name of pregnant woman: | Identification number: | National code: |
| --- | --- | --- | --- |
| Interviewer code: | Last name of questioner: | Name of interviewer: | Date of interview: |
| Address: | | Phone or mobile number of woman: | |

1. **Demographic and basic information**
2. Can the woman answer the questions? Yes (Please go back to question 4 ) No 
3. Why can't the woman answer the questions?

Inappropriate physical condition ٱ

Inappropriate mental health status ٱ

Different language of questioner ٱ

Refusal or unwillingness to interview ٱ

1. Please identify the responsive relationship with woman.

Spouse ٱ

Child ٱ

Child of spouse ٱ

Granddaughter ٱ

Father or mother ٱ

Father or mother of spouse ٱ

Sister or brother ٱ

Second wife ٱ

Stepchild ٱ

Other relative’s ٱ

Non-relative ٱ

Unknown ٱ

1. What is your educational status?

Illiterate ٱ

Primary (incomplete) ٱ

Primary (complete) ٱ

Guidance (complete) ٱ

Guidance (incomplete) ٱ

High school (incomplete) ٱ

Diploma ٱ

Academic ٱ

1. What is your spouse's educational status?

Illiterate ٱ

Primary (incomplete) ٱ

Primary (complete) ٱ

Guidance (complete) ٱ

Guidance (incomplete) ٱ

High school (incomplete) ٱ

Diploma ٱ

Academic ٱ

1. What was your employment status during the past 12 months?

Governmental Employment ٱ

Non-Governmental ٱ

Employment ٱ

Self-employed ٱ

Voluntary work (without wage) ٱ

Student ٱ

Housewife ٱ

Retired ٱ

Unemployed (job seekers) ٱ

Unemployed (disabled) ٱ

Daily pay job/ seasonal employment ٱ

1. What was your spouse’s job during the past 12 months?

Governmental Employment ٱ

Non-Governmental Employment ٱ

Self-employed ٱ

Voluntary work (without wage) ٱ

Student ٱ

Retired ٱ

Unemployed (job seekers) ٱ

Unemployed (disabled) ٱ

Daily pay job/ seasonal employment ٱ

1. Are you covered by a health insurance? Yes ٱ No ٱ
2. Which health insurance coverage do you have? (You can select more than one option)

Medical services ٱ

Social security ٱ

Armed forces ٱ

Imam Khomeini relief committee ٱ

Other insurance ٱ (Please mention) ............ 

I do not know ٱ

1. Are you covered by complementary insurance?

Yes ٱ ٱ (insurance type): …

No ٱ

I do not know ٱ

1. **Determining the quality of life of pregnant mother**

The following questions are about your current health status. Please specify the best option that represents your health status currently.

1. Moving around

I have no problem for moving around in my surroundings ٱ

I have some difficulty in moving around in my surroundings ٱ

I have become crippled and I am unable move around in my environment ٱ

1. Self-care

I have no problem to take care of myself ٱ

I have some problems with my washing and changing clothes ٱ

I am unable to wash or change my clothes ٱ

1. Routine activities

I have no problems with my usual activities (work, study, homework, family affairs and leisure) ٱ

I have some problems with my usual chores ٱ

I am unable to do my usual activities ٱ

1. Pain / discomfort

I have no pain or discomfort ٱ

I have somewhat pain and discomfort ٱ

I have too much pain and discomfort ٱ

1. Anxiety / Depression

I'm not anxious or depressed ٱ

I'm somewhat anxious or depressed ٱ

I'm extremely anxious or depressed ٱ

1. Now, I want you to tell me what you think about your health status today. To help you, we have plotted a thermometer-like scale for you on this page. On this scale, the best imaginable health condition is equivalent to 100, and the worst case is zero. Please comment on this by drawing an arrow on the left side of the figure that represents your health at the moment.

**Responsive health status**

|  | The best imaginable health condition | | | |
| --- | --- | --- | --- | --- |
|  |  |  |  |  |
|  | 100 | | | |
|  |  |  |  |  |
|  |  |  |  |  |
|  |  |  |  |  |
|  |  |  |  |  |
|  |  |  |  |  |
|  |  |  |  |  |
|  |  |  |  |  |
|  |  |  |  |  |
| 90 |  |  |  |  |
|  |  |  |  |  |
|  |  |  |  |  |
|  |  |  |  |  |
|  |  |  |  |  |
|  |  |  |  |  |
|  |  |  |  |  |
|  |  |  |  |  |
|  |  |  |  |  |
|  |  |  |  |  |
| 80 |  |  |  |  |
|  |  |  |  |  |
|  |  |  |  |  |
|  |  |  |  |  |
|  |  |  |  |  |
|  |  |  |  |  |
|  |  |  |  |  |
|  |  |  |  |  |
|  |  |  |  |  |
|  |  |  |  |  |
| 70 |  |  |  |  |
|  |  |  |  |  |
|  |  |  |  |  |
|  |  |  |  |  |
|  |  |  |  |  |
|  |  |  |  |  |
|  |  |  |  |  |
|  |  |  |  |  |
|  |  |  |  |  |
|  |  |  |  |  |
| 60 |  |  |  |  |
|  |  |  |  |  |
|  |  |  |  |  |
|  |  |  |  |  |
|  |  |  |  |  |
|  |  |  |  |  |
|  |  |  |  |  |
|  |  |  |  |  |
|  |  |  |  |  |
|  |  |  |  |  |
| 50 |  |  |  |  |
|  |  |  |  |  |
|  |  |  |  |  |
|  |  |  |  |  |
|  |  |  |  |  |
|  |  |  |  |  |
|  |  |  |  |  |
|  |  |  |  |  |
|  |  |  |  |  |
|  |  |  |  |  |
| 40 |  |  |  |  |
|  |  |  |  |  |
|  |  |  |  |  |
|  |  |  |  |  |
|  |  |  |  |  |
|  |  |  |  |  |
|  |  |  |  |  |
|  |  |  |  |  |
|  |  |  |  |  |
|  |  |  |  |  |
| 30 |  |  |  |  |
|  |  |  |  |  |
|  |  |  |  |  |
|  |  |  |  |  |
|  |  |  |  |  |
|  |  |  |  |  |
|  |  |  |  |  |
|  |  |  |  |  |
|  |  |  |  |  |
|  |  |  |  |  |
| 20 |  |  |  |  |
|  |  |  |  |  |
|  |  |  |  |  |
|  |  |  |  |  |
|  |  |  |  |  |
|  |  |  |  |  |
|  |  |  |  |  |
|  |  |  |  |  |
|  |  |  |  |  |
|  |  |  |  |  |
| 10 |  |  |  |  |
|  |  |  |  |  |
|  |  |  |  |  |
|  |  |  |  |  |
|  |  |  |  |  |
|  |  |  |  |  |
|  |  |  |  |  |
|  |  |  |  |  |
|  |  |  |  |  |
|  |  |  |  |  |
|  |  |  |  |  |
|  |  |  |  |  |
|  | 0 | | | |
|  |  |  |  |  |
|  |  |  |  |  |
|  | The worst imaginable health condition | | | |
|  |  |  |  |  |
|  |  |  |  |  |
|  |  |  |  |  |
|  |  |  |  |  |
|  |  |  |  |  |
|  |  |  |  |  |

**Maternal costs**

**The cost of maternal care during pregnancy**

1. Which of the following hospital personnel (specialist, technicians, etc.) have visited you during last month for prenatal care?

Midwife ------ times

General practitioner ------ times

Gynecologist ------ times

Internal medicine specialist / endocrinologist ------ times

Ultrasonography ------ times

Laboratory ------ times

1. How much time does the midwife spend with you at each visit?

Beside you from home to the clinic (service delivery unit) ... min

Appointment ... min

Waiting time (service delivery unit) … min

Access to Health Services (service delivery unit) … min

1. How much time does the general practitioner spend with you at each visit?

Beside you from home to the clinic (service delivery unit) ... min

Appointment ... min

Waiting time (service delivery unit) … min

Access to Health Services (service delivery unit) … min

1. How much time does the gynecologist spend with you at each visit?

Beside you from home to the clinic (service delivery unit) ... min

Appointment ... min

Waiting time (service delivery unit) … min

Access to Health Services (service delivery unit) … min

1. How much time does the internal medicine specialist / endocrinologist spend with you at each visit?

Beside you from home to the clinic (service delivery unit) ... min

Appointment ... min

Waiting time (service delivery unit) … min

Access to Health Services (service delivery unit) … min

1. How much time do you spend to have ultrasonography/ biophysical profiles at each visit?

Beside you from home to the clinic (service delivery unit) ... min

Appointment ... min

Waiting time (service delivery unit) … min

Access to Health Services (service delivery unit) … min

1. How much time is spend having your laboratory tests done?

Beside you from home to the clinic (service delivery unit) ... min

Appointment ... min

Waiting time (service delivery unit) … min

Access to Health Services (service delivery unit) … min

1. How much money do you pay for each visit by midwife to get care?

Travel to the service delivery unit … Rials

Payment for visit cost … Rials

Cost of medicine … Rials

1. How much money do you pay the general practitioner for each visit?

Travel to the service delivery unit … Rials

Payment for visit cost … Rials

Cost of medicine … Rials

1. How much money do you pay the gynecologist for each visit?

Travel to the service delivery unit … Rials

Payment for visit cost … Rials

Cost of medicine … Rials

1. How much money do you pay the internal medicine specialist / endocrinologist for each visit?

Travel to the service delivery unit … Rials

Payment for visit cost … Rials

Cost of medicine … Rials

1. How much money do you pay for each ultrasonography each time?

Travel to the service delivery unit … Rials

Payment for visit cost … Rials

Cost of medicine … Rials

1. How much money do you pay for your laboratory tests each time?

Travel to the service delivery unit … Rials

Payment for visit cost … Rials

Cost of medicine … Rials

1. How much time do you have to take off (job) for each visit to the midwife each time?

… Hour ….. Day Not employed

1. How much time do you have to take off (job) for each visit to the general practitioner each time?

… Hour ….. Day Not employed

1. How much time do you have to take off (job) for each visit to the gynecologist each time?

… Hour ….. Day Not employed

1. How much time do you have to take off (job) for each visit to the internal medicine specialist / endocrinologist each time?

… Hour ….. Day Not employed

1. How much time do you have to take off (job) for each sonography?

… Hour ….. Day Not employed

1. How much time do you have to take off (job) to be performed laboratory tests each time?

… Hour ….. Day Not employed

1. Does anyone accompany you for the doctors? Yes ٱ Noٱ
2. Who accompanies you? Relationship of accompanying person with woman … Name: …
3. If you have gestational diabetes, do you use a glucometer to measure your blood glucose at home?

Yes, I regularly measure my blood glucose.

Yes, I have a glucometer, but I do not regularly measure my blood glucose.

Yes, I have a glucose meter, but I cannot afford its strips and solutions, and hence I do not use it regularly.

No, I do not have a glucometer.

1. What is the average cost of using a glucometer for duration of a month? … Rials

**Medical services related to the hospitalization of the pregnant mother**

Now, I want to ask you questions about your admission to the hospital during pregnancy. The purpose of admission to a hospital is that you are not discharged or referred immediately elsewhere after the initial examination or evaluation; therefore, you are monitor for several hours at least.

1. Have you been hospitalized last month? Yes ٱ Noٱ
2. How many times have you been hospitalized in the last month?

.... times

I do not know

| Number of hospitalizations | Cause of hospitalizations | Related to pregnancy | Unrelated to pregnancy | Type of hospital   1. Governmental 2. Charity 3. I don’t know | Number of days hospitalized | Insurance coverage   1. Medical services 2. Social security 3. Armed forces 4. Imam Khomeini relief committee | Complementary insurance coverage   1. Yes 2. No | How much money did you pay to the hospital?  (Rial) | Out-of-hospital expenses (drug purchase, medical supplies, etc.) |
| --- | --- | --- | --- | --- | --- | --- | --- | --- | --- |
|  |  |  |  |  |  |  |  |  |  |
|  |  |  |  |  |  |  |  |  |  |
|  |  |  |  |  |  |  |  |  |  |

**Childbirth**

Now, I want to ask you questions about your admission to the hospital for childbirth. The purpose of admission to a hospital is that you do not discharge or refer immediately after the initial examination or evaluation; therefore, you monitor for at least several hours.

1. Have you been hospitalized for delivery? Yesٱ Noٱ
2. What kind of hospital have you been hospitalized?

Private ٱ

Governmental ٱ

Charity ٱ

I do not know ٱ

1. Did the hospital accept your insurance?

Yes ٱ

No ٱ

I have no insurance ٱ

I do not know ٱ

1. If yes, which type of insurance? (You can select more than one option)

Medical services ٱ

Social security ٱ

Armed forces ٱ

Imam Khomeini relief committee ٱ

Complementary insurance ٱ

1. How many days were you hospitalized? … days
2. Are you admitted to ICU or CCU? Yes ٱ ……….. days No
3. How much money did you pay for hospitalization expenses?

… Rials

Refuse ٱ

I do not know ٱ

1. How much money did you spend outside the hospital (buying medicines, medical supplies, etc.)?

… Rials

Refuse ٱ

I do not know ٱ

**Neonatal Costs**

Now, I want to ask you questions about your neonate admission to the hospital for childbirth. The purpose of admission to a hospital is that you do not discharge or refer immediately after the initial examination or evaluation; therefore, you monitor for at least several hours.

1. Has the baby stayed at the hospital after discharge of the mother? Yes ٱ No ٱ
2. How many times have your baby been admitted to the hospital after birth?  … times

| Number of hospitalizations | Cause of hospitalizations | Related to pregnancy | Unrelated to pregnancy | Type of hospital  Governmental  Charity  I don’t know | The number of days hospitalized | Insurance coverage  Medical services  Social security  Armed forces  Imam Khomeini relief committee | Supplementary insurance coverage  Yes  No | How much money did you pay to the hospital?  (Rials) | Out-of-hospital expenses (drug purchase, medical supplies, etc.) |
| --- | --- | --- | --- | --- | --- | --- | --- | --- | --- |
|  |  |  |  |  |  |  |  |  |  |
|  |  |  |  |  |  |  |  |  |  |
|  |  |  |  |  |  |  |  |  |  |
